# A Colloid Cyst Masquerading As Idiopathic Intracranial Hypertension in a Young Woman: A Diagnostic Pitfall

**DOI:** 10.7759/cureus.95584

**Published:** 2025-10-28

**Authors:** Dylon J Wetzel, Kathryn Boswell

**Affiliations:** 1 College of Medicine, Florida State University College of Medicine, Tallahassee, USA; 2 Department of Emergency Medicine, Orlando Regional Medical Center, Orlando, USA

**Keywords:** colloid cyst, endoscopic resection, headache, idiopathic intracranial hypertension, neuroimaging, obstructive hydrocephalus, ocular ultrasound, papilledema, third ventricle, visual obscuration

## Abstract

Colloid cysts of the third ventricle are rare, benign intracranial lesions that can produce a broad spectrum of symptoms that depend on both the degree and rate of cerebrospinal fluid (CSF) obstruction. When the presentation is insidious, these lesions may at first mimic idiopathic intracranial hypertension (IIH), particularly in young women who may first develop headaches and papilledema without any focal neurological deficits on exam. We describe the case of a 24-year-old woman with several months of non-positional headaches accompanied by intermittent nausea, photophobia, and brief episodes of visual obscuration. Initially, she was evaluated by an optometrist, who identified bilateral papilledema and further recommended Diamox (acetazolamide) and brain imaging. Upon presentation to the emergency department, her neurological examination was normal, but the bedside ocular ultrasound (US) demonstrated optic nerve sheath enlargement and optic disc elevation that is consistent with papilledema. The non-contrast computed tomography (CT) of the head then revealed a 1.3 x 1.4 x 1.8 cm hyperdense lesion at the foramen of Monro, causing obstructive hydrocephalus, consistent with a colloid cyst. As such, the patient was admitted to the neuro-intensive care unit for monitoring and underwent endoscopic resection of the cyst. Her postoperative recovery was relatively uncomplicated. Furthermore, her headaches and visual symptoms were resolved completely. This case illustrates how a slowly progressive colloid cyst can masquerade as IIH and highlights the importance of obtaining neuroimaging prior to lumbar puncture in any patient presenting with papilledema. Maintaining a broad differential that recognizes secondary causes of elevated intracranial pressure early is essential to prevent vision loss and ensure timely neurosurgical management.

## Introduction

Colloid cysts of the third ventricle are rare, benign, epithelial-lined lesions that account for approximately 0.5%-1% of all intracranial tumors [1]. Their clinical presentation generally varies widely depending on the size of the cyst and the degree of cerebrospinal fluid (CSF) obstruction [2]. While acute obstruction of CSF flow can lead to rapid neurological decline and even sudden death [3], slowly enlarging cysts may present with only subtle or nonspecific symptoms. Because the foramen of Monro is the small passageway that allows CSF to flow from each lateral ventricle into the third ventricle, a cyst here can block that pathway and lead to fluid buildup and pressure in the brain. As such, diagnosis can be delayed or confounded by overlapping features with other causes of increased intracranial pressure [2,3].

Idiopathic intracranial hypertension (IIH) is a disorder characterized by elevated intracranial pressure without an identifiable intracranial mass or hydrocephalus (accumulation of fluid within the brain’s ventricles) [4]. This typically affects young women, producing headaches, transient visual obscurations, and papilledema (optic-disc swelling due to raised intracranial pressure) [4]. Diagnostic and management guidelines further emphasize this demographic pattern [5]. Patients with both IIH and colloid cysts can present with headaches and visual disturbances in similar demographics [4,5], in which diagnostic confusion has been well documented in recent literature [6]. Thus, distinguishing between them promptly is critical, especially since their management differs drastically.

We present the case of a young woman whose gradually progressive headaches and papilledema were initially suggestive of IIH but were ultimately found to be due to a colloid cyst at the foramen of Monro causing obstructive hydrocephalus. This case highlights the importance of maintaining a broad differential diagnosis for patients presenting with papilledema and further emphasizes the need for neuroimaging before performing a lumbar puncture to exclude secondary causes of raised intracranial pressure [4-6]. Other conditions producing similar findings to consider for a broadened differential include cerebral venous sinus thrombosis, intracranial tumors, meningitis, and medication-related intracranial hypertension.

## Case presentation

A 24-year-old woman with no significant past medical history presented to the emergency department with several months of progressively worsening, non-positional headaches. She described the headaches as dull and pressure-like, occasionally associated with nausea, vomiting, photophobia, and brief episodes of visual obscuration. The visual obscurations typically lasted several minutes and were most noticeable upon rising from a reclined position. However, she reported no diplopia, focal weakness, numbness, changes in consciousness, or other neurological symptoms.

The patient had initially sought evaluation from an optometrist due to the persistence of her headaches and blurred vision despite over-the-counter remedies. Her fundoscopic examination (a visual inspection of the retina and optic disc in the back of the eye) then revealed bilateral papilledema, and they told her they were concerned she may have pseudotumor cerebri (another term synonymous with IIH). The optometrist recommended initiation of acetazolamide (Diamox), a carbonic anhydrase inhibitor used to reduce CSF production and lower intracranial pressure, as well as obtaining further neuroimaging with MRI, prompting the patient to seek emergency medical evaluation.

**Figure 1:**
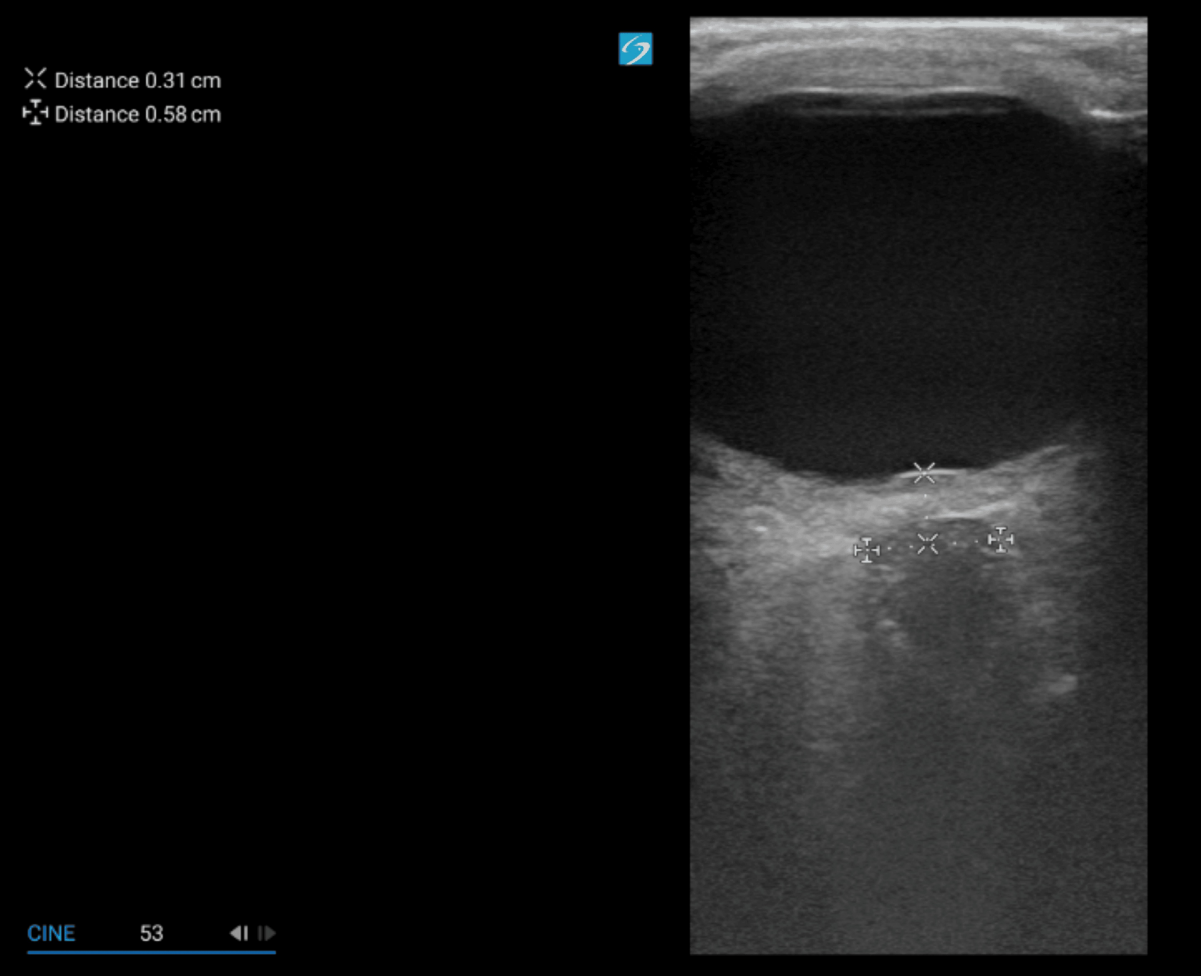
The optic nerve sheath diameter (0.58 cm) measured approximately 3 mm posterior to the retina.

**Figure 2:**
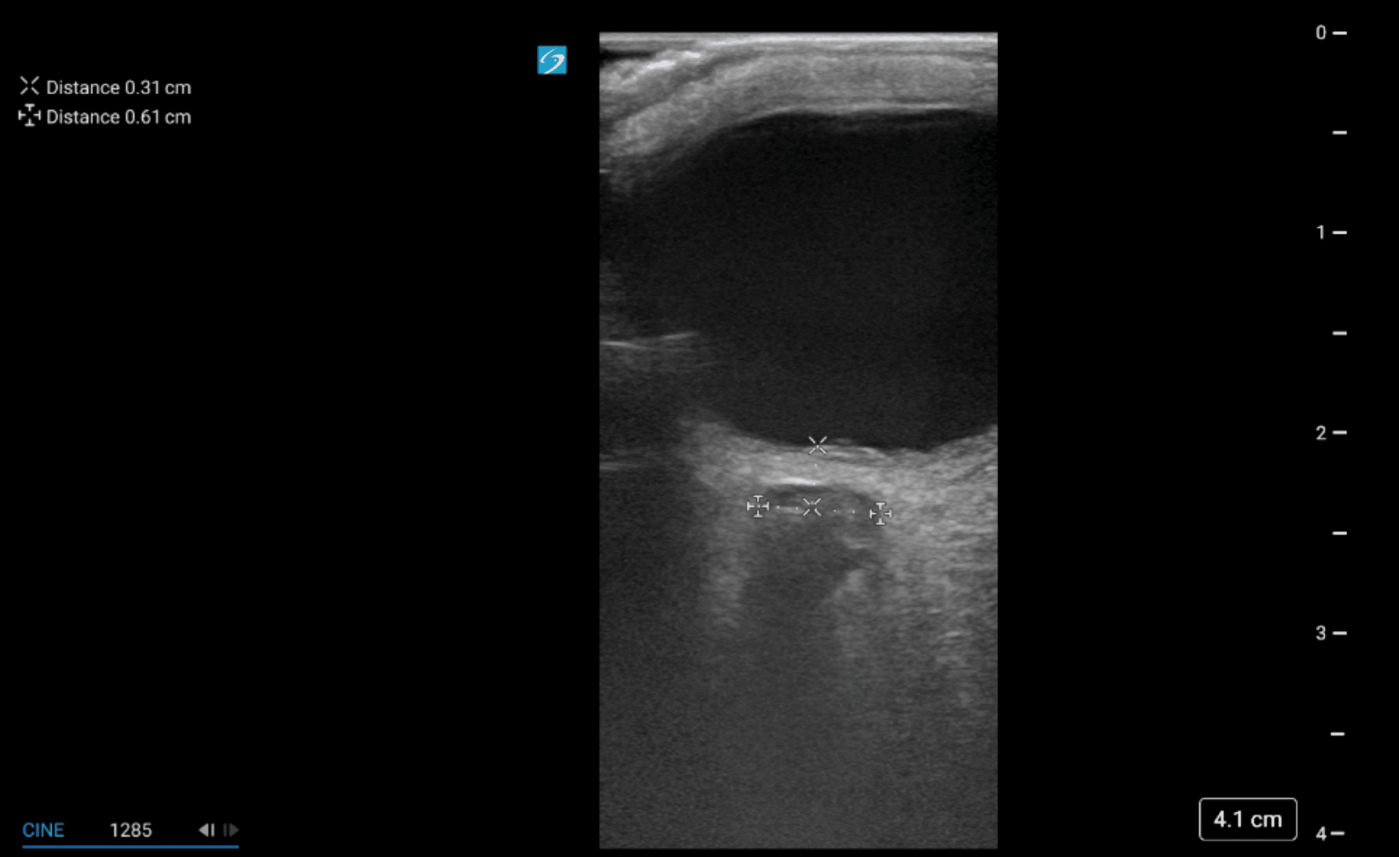
The contralateral eye showed an optic nerve sheath diameter of 0.61 cm, confirming bilateral findings consistent with raised intracranial pressure.

**Figure 3:**
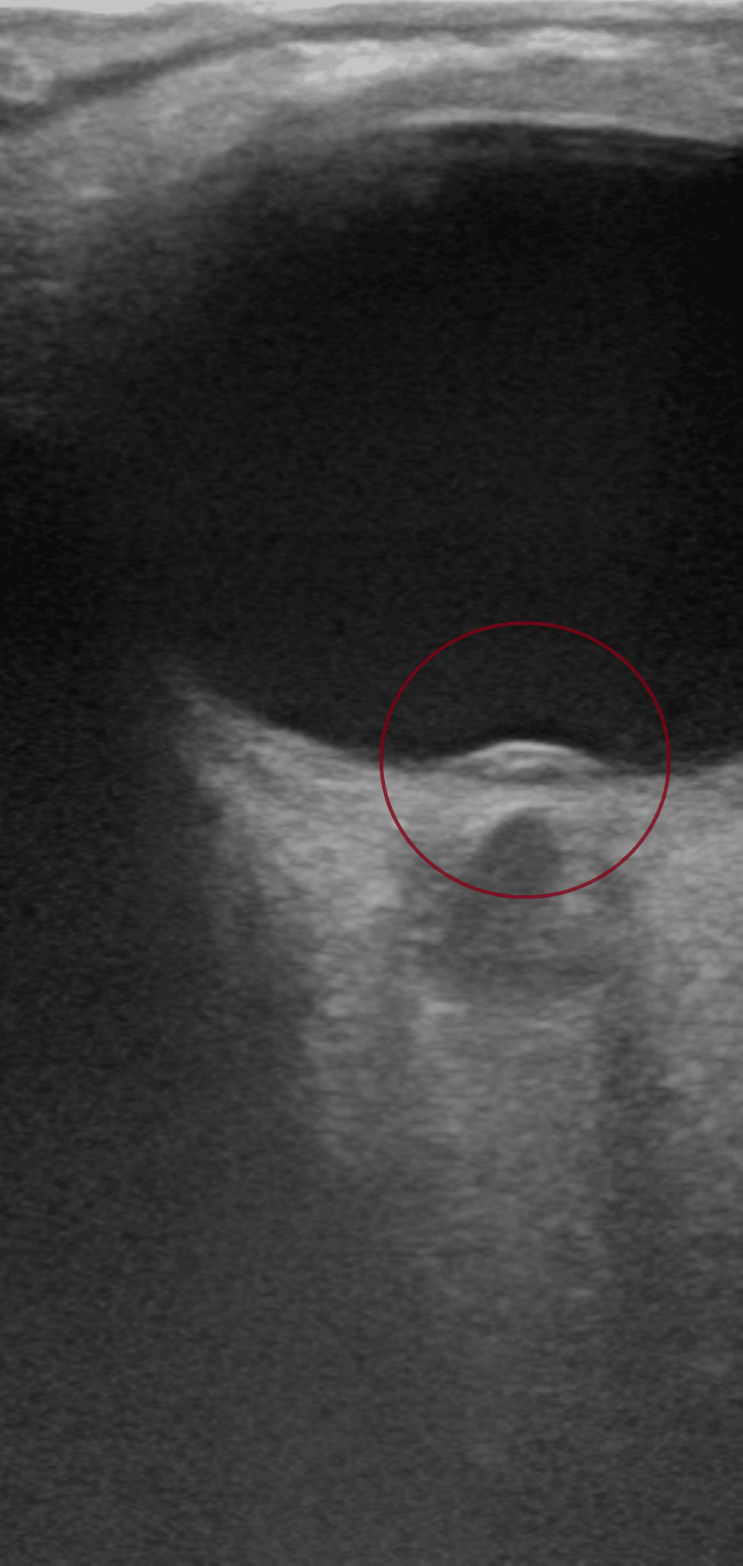
Optic disc elevation (papilledema) protruding into the vitreous cavity, visualized as a convex disc bulge (circled in red).

Upon arrival to the emergency department, her triage vital signs were stable within normal limits (blood pressure 133/86 mmHg, heart rate 98 bpm, respiratory rate 17/min, temperature 36.8°C, and oxygen saturation 97% on room air). Her body mass index was noted to be 28.7 kg/m². Neurological examination was non-focal, with intact cranial nerves, motor strength, and sensation. A bedside ocular ultrasound (US) was then performed, revealing optic nerve sheath enlargement and elevation of the optic disc consistent with papilledema (Figures 1-3).

**Figure 4:**
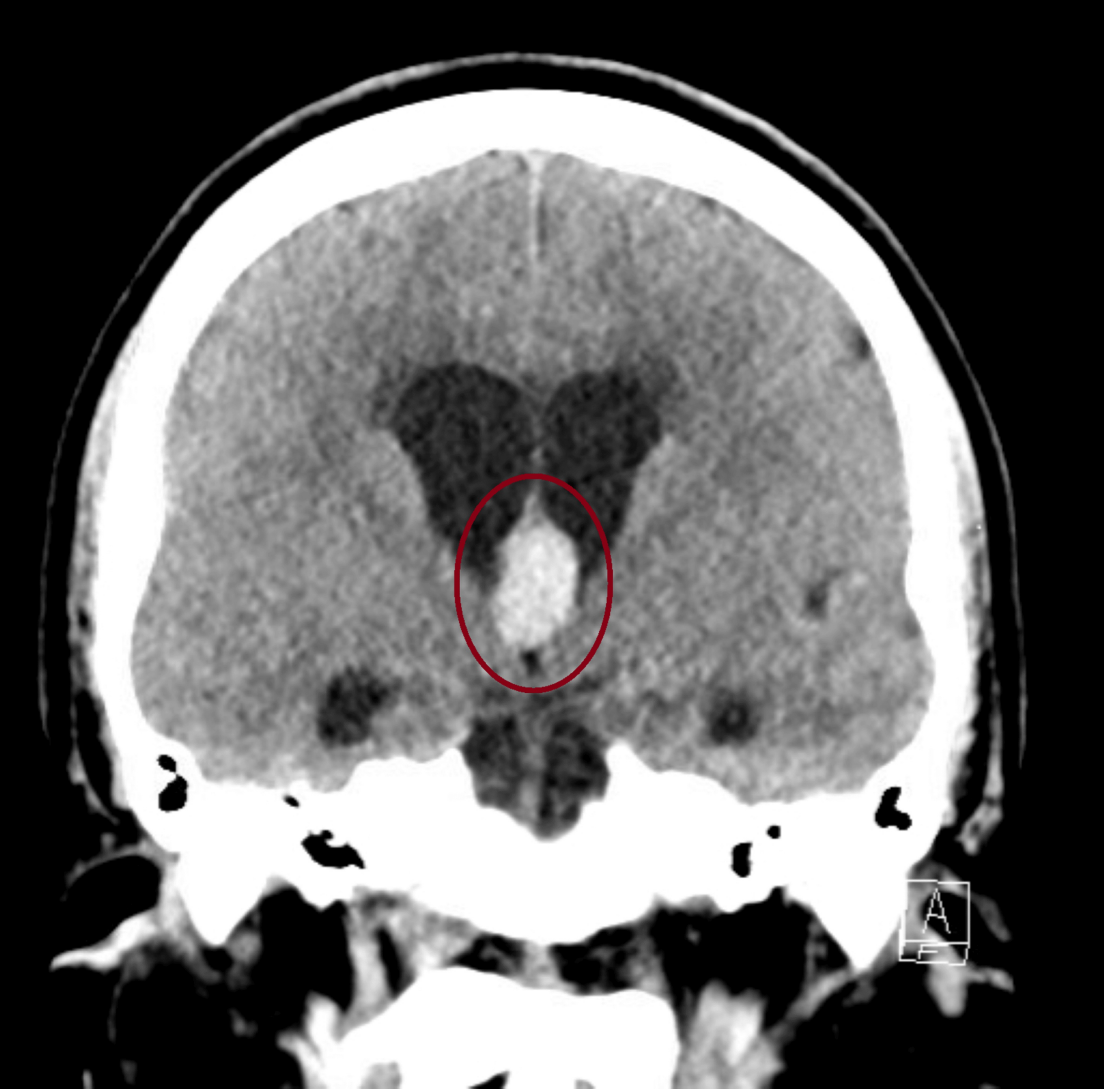
Axial non-contrast CT showing a hyperdense colloid cyst at the foramen of Monro with associated ventricular enlargement.

**Figure 5:**
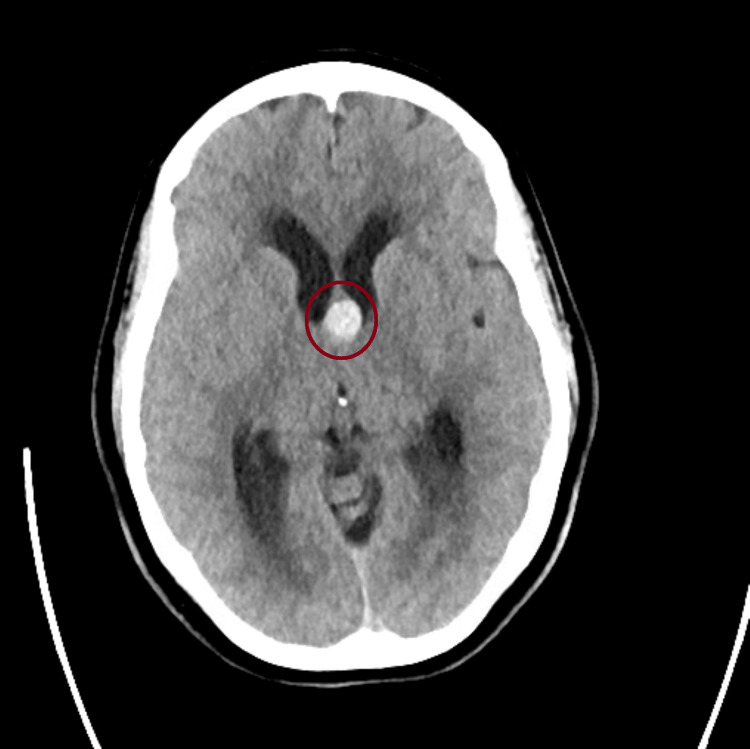
Coronal non-contrast CT showing a hyperdense colloid cyst at the foramen of Monro with associated ventricular enlargement.

A non-contrast computed tomography (CT) scan of the head demonstrated a 1.3 x 1.4 x 1.8 cm hyperdense lesion at the foramen of Monro obstructing the foramen and resultant dilatation of the lateral ventricles, consistent with obstructive hydrocephalus (Figures 4, 5).

**Figure 6:**
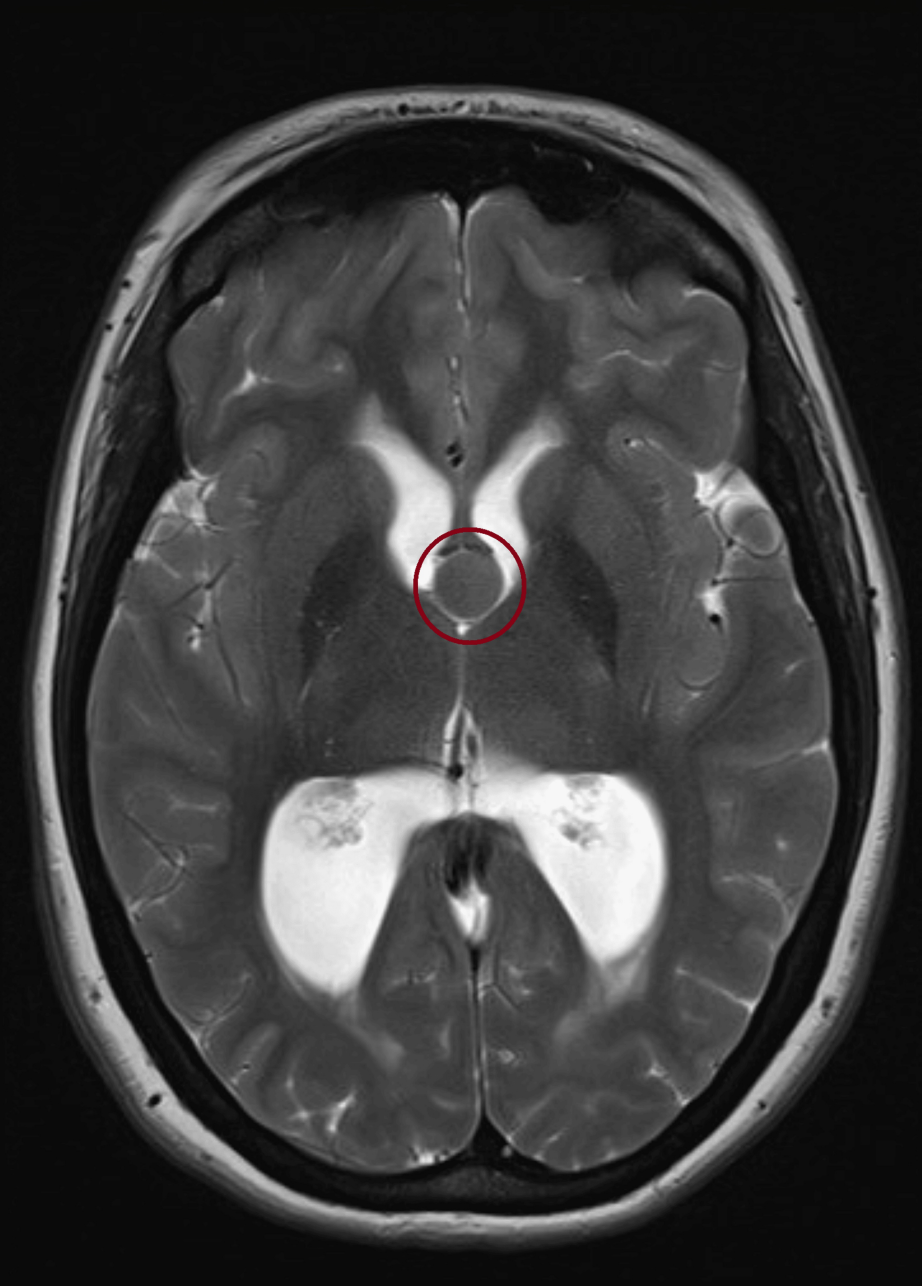
Axial T1-weighted MRI demonstrating a hyperintense, well-defined colloid cyst at the foramen of Monro with associated ventricular dilation.

**Figure 7:**
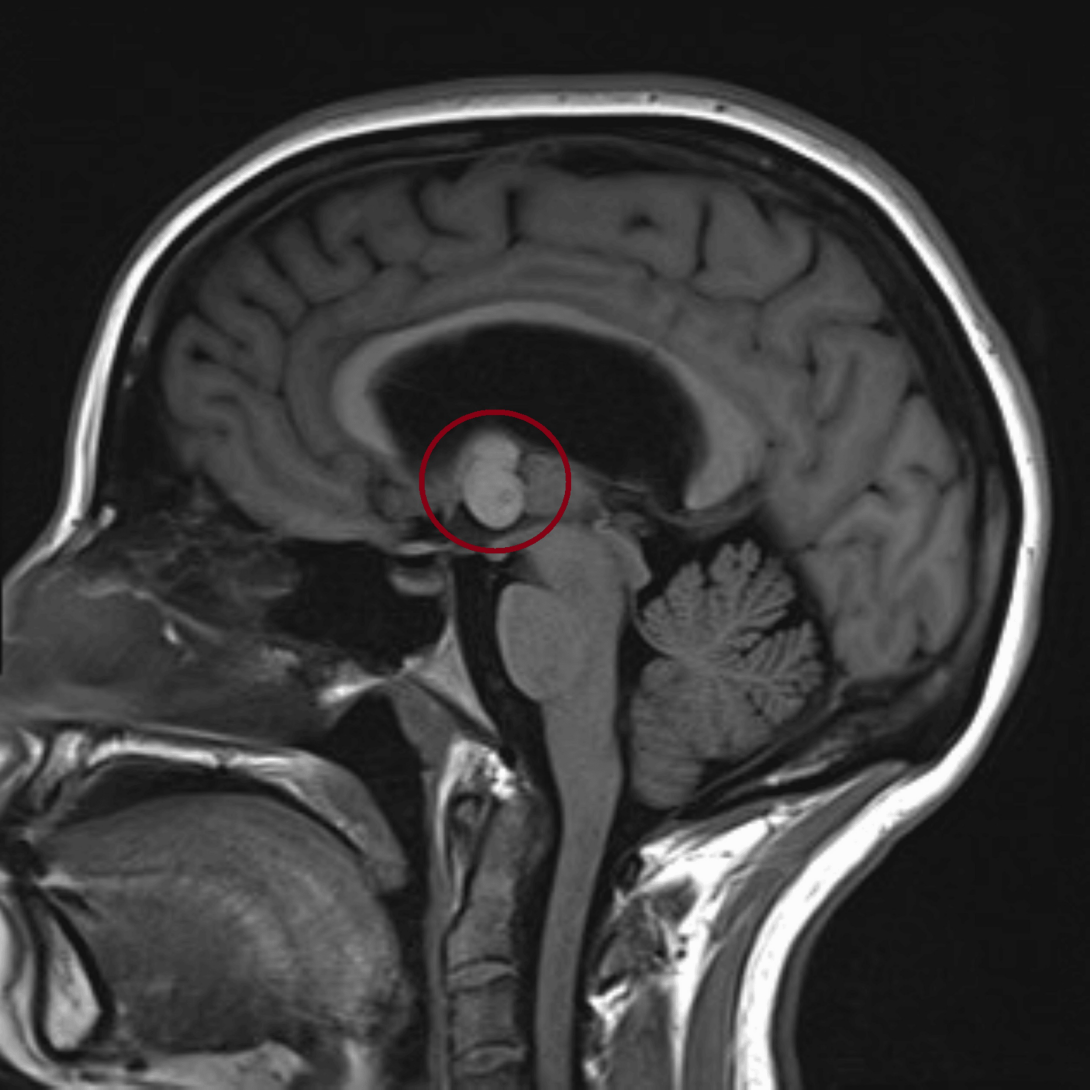
Sagittal T1-weighted MRI demonstrating a hyperintense, well-defined colloid cyst at the foramen of Monro with associated ventricular dilation.

Magnetic resonance imaging (MRI) was done after, and further characterized the lesion as a well-circumscribed, non-enhancing, T1 hyperintense and T2 hypointense cyst at the anterior third ventricle, compatible with a colloid cyst (Figures 6, 7).

She was then admitted to the neurosurgical service for close monitoring in the neuro-intensive care unit. Overnight, she remained neurologically stable without signs of acute decompensation. The next day, endoscopic resection of the colloid cyst with septum pellucidotomy (a procedure that creates a small opening in the thin membrane, septum pellucidum, separating the lateral ventricles) was performed, and a temporary external ventricular drain (EVD) was placed intraoperatively for CSF diversion.

Postoperatively, her headaches and visual disturbances resolved completely. She experienced no complications or evidence of CSF leak. After several days, the EVD was successfully removed, after which she was discharged home in a stable condition with a referral for outpatient neurosurgery follow-up. She returned to the clinic for her two-week postoperative visit, noting that she remained asymptomatic without recurrence of headaches or visual disturbances with a full return to baseline functioning.

## Discussion

This case highlights how a slowly progressive colloid cyst can look like IIH at first in a young woman with headaches and papilledema but no focal deficits, and how bedside ocular US can push clinicians toward urgent neuroimaging that changes management. In our patient, optic nerve sheath enlargement and disc elevation on point-of-care US (POCUS) supported raised intracranial pressure at the bedside, and the subsequent CT/MRI identified a third-ventricular colloid cyst obstructing the foramen of Monro. This sequence underscores why it is important to keep the differential diagnosis process broad in scope when evaluating papilledema and not assume IIH up front [4-6].

Colloid cysts are benign, epithelial-lined lesions of the third ventricle that account for roughly 0.5%-1% of intracranial tumors and classically arise near the foramen of Monro [1-3]. Depending on size, position, and mobility, they can intermittently or persistently impede CSF flow and produce symptoms that evolve from positional headaches to more constant, nonspecific complaints; in rare circumstances, acute obstructive hydrocephalus or even sudden deterioration can occur [1-3]. This overlap with IIH of headache, papilledema, and transient visual obscurations can lead to anchoring/confirmation bias and delayed recognition of a structural cause [4-6]. In our patient, that overlap was present at first, but the imaging clarified the diagnosis.

Per the modified Dandy criteria, IIH requires elevated intracranial pressure with normal neuroimaging (no mass lesion and no hydrocephalus) [4,5]. Because our patient’s MRI showed ventricular enlargement and an obstructing third-ventricular cyst, she did not meet IIH criteria. This is why neuroimaging should precede lumbar puncture in any patient with papilledema, both to avoid herniation risk and to identify secondary causes such as mass lesions, venous sinus thrombosis, or hydrocephalus when present [4-6].

POCUS was useful here. Visualization of optic nerve sheath dilation and optic disc elevation provided a quick, noninvasive signal of raised intracranial pressure and helped triage the patient to urgent CT/MRI [7,8]. While ocular US cannot distinguish IIH from obstructive causes, it can validate suspected intracranial hypertension at the bedside and accelerate definitive imaging and consultation in the emergency setting [7,8]. This is exactly how it functioned in our case.

Imaging then defined the lesion: a 1.3 × 1.4 × 1.8 cm colloid cyst at the foramen of Monro with bilateral ventricular enlargement and transependymal CSF flow, explaining the patient’s symptoms and exam findings [1-3]. For symptomatic cysts or those causing hydrocephalus, surgery remains the preferred treatment [1,3,9]. Our patient underwent endoscopic resection with septum pellucidotomy and temporary EVD placement for CSF diversion. A septum pellucidotomy means creating a small opening in the thin membrane (septum pellucidum) that separates the lateral ventricles to improve CSF communication and reduce the chance of postoperative obstruction [9]. Endoscopic techniques offer direct visualization and safe excision with lower morbidity than traditional open approaches in appropriate candidates [1,9].

Postoperatively, her headaches and visual symptoms resolved, and she remained asymptomatic at follow-up, which is consistent with published reports after endoscopic removal [1,3,9]. Endoscopic resection has shown high gross-total removal rates, meaning complete excision of the cyst and its capsule under endoscopic visualization, with reported recurrence rates below five percent [9]. Although rare, recurrences have been documented even years after surgery, particularly when small portions of the cyst wall remain, so continued long-term follow-up is advised [3,9]. A practical plan is outpatient neurosurgery follow-up with periodic MRI, possibly every one to two years, to monitor for recurrence and to track ventricular size over time [9]. This approach supports not only neurological safety but also preservation of quality of life by preventing a delayed return of hydrocephalus-related symptoms.

Two important clinical lessons are highlighted by this case. First, slowly developing colloid cysts can masquerade as IIH in young women and should be part of a wide array of elements in the differential diagnosis whenever papilledema is present [4-6]. Second, ocular US in the emergency setting can screen quickly for papilledema, justify immediate neuroimaging, and help direct timely neurosurgical consultation when indicated [7,8]. Applying this stepwise, broad approach helps prevent vision loss, neurological decline, and unnecessary procedures, and it supports faster return to baseline functioning [1,3,5].

## Conclusions

This case highlights how a colloid cyst can closely mimic idiopathic IIH and the importance of maintaining a broad and careful diagnostic approach. It is crucial to include a wide array of elements in the differential diagnosis, including structural causes like colloid cysts, even when the presentation seems typical for IIH. Keeping the differential diagnosis process broad in scope helps avoid overlooking serious underlying conditions and ensures timely intervention. Bedside ocular ultrasound can be a quick and valuable tool for confirming papilledema and guiding early imaging, which can be lifesaving in the right context. With appropriate neuroimaging and surgical management, patients can make a full recovery and avoid major complications. Beyond preventing vision loss or early deterioration, prompt diagnosis and treatment also play a key role in preserving long-term quality of life.
